# Imported Visceral Leishmaniasis in Timiș County, Western Romania

**DOI:** 10.3390/microorganisms14061196

**Published:** 2026-05-26

**Authors:** Alin Gabriel Mihu, Ioana Ionita, Mariana Patiu, Coralia Adina Cotoraci, Daniela Adriana Oatis, Doina Elena Nicola, Ana Alexandra Ardelean, Liana Maria Chicea, Tudor Rareș Olariu

**Affiliations:** 1 Center for Diagnosis and Study of Parasitic Diseases, Department of Infectious Disease, “Victor Babes” University of Medicine and Pharmacy, 300041 Timisoara, Romania; alin.mihu@umft.ro (A.G.M.); daniela.oatis@umft.ro (D.A.O.); rolariu@umft.ro (T.R.O.); 2 Department of Biology and Life Sciences, Vasile Goldis Western University of Arad, 310300 Arad, Romania; 3 Patogen Preventia, 300124 Timisoara, Romania; 4 Department of Internal Medicine, University Clinic of Hematology, “Victor Babes” University of Medicine and Pharmacy, No. 2 Eftimie Murgu Square, 300041 Timisoara, Romania; ionita.ioana@umft.ro; 5 Multidisciplinary Research Center for Malignant Hemopathies, “Victor Babes” University of Medicine and Pharmacy, Eftimie Murgu Square 2, 300041 Timisoara, Romania; 6 Department of Hematology, Ion Chiricuta Oncology Institute, 400015 Cluj-Napoca, Romania; mpatiu@yahoo.com; 7 Department of Medicine, Vasile Goldis Western University of Arad, 310025 Arad, Romania; rector_vg@uvvg.ro; 8 Clinical Laboratory, Municipal Clinical Emergency Teaching Hospital, 300254 Timisoara, Romania; doinanicola@gmail.com; 9 Discipline of Parasitology, Department of Infectious Disease, “Victor Babes” University of Medicine and Pharmacy, 300041 Timisoara, Romania; 10 Department II Medical Clinic, “Victor Papilian” Faculty of Medicine, Lucian Blaga University of Sibiu, 550024 Sibiu, Romania; 11 Internal Medicine Department, Academic Emergency Hospital, 550245 Sibiu, Romania

**Keywords:** *Leishmaniasis*, *Leishmania*, Romania, bone marrow aspirate, pancytopenia, splenomegaly, atypical

## Abstract

Visceral leishmaniasis (VL) is a rare but increasingly recognized parasitic infection in non-endemic European regions, including Romania. We report two cases of VL diagnosed in Timiș County, Western Romania, in 2020: a 57-year-old male presenting with fever, massive splenomegaly, and severe pancytopenia, and a 51-year-old male presenting atypically with fatigue, bicytopenia, and absence of organomegaly. Both patients were diagnosed through bone marrow aspirate examination revealing *Leishmania* amastigotes. Both cases were classified as imported VL, with exposure linked to endemic regions of eastern Spain (Castellón and València). These cases highlight the importance of maintaining a high index of clinical suspicion for VL in patients presenting with unexplained cytopenias and elevated inflammatory markers, even in the absence of classic features such as hepatosplenomegaly. The present report underscores the relevance of travel history in non-endemic regions such as Romania and the need for clinical awareness of imported VL in patients returning from endemic areas of southern Europe.

## Simple Summary

Visceral leishmaniasis is a serious parasitic disease transmitted by sandflies, rare in Romania but common in parts of southern Europe such as Spain. We describe two adult male patients from Western Romania who developed the infection after traveling to endemic regions of eastern Spain. One showed the classic presentation (fever, splenomegaly, and pancytopenia) while the other had only mild, atypical symptoms with no organomegaly. In both, the diagnosis was confirmed by bone marrow examination. These cases highlight the need to consider this potentially fatal disease in travelers, even when symptoms are atypical.

## 1. Introduction

Leishmaniasis is a significant vector-borne disease caused by over 20 *Leishmania* species and transmitted by phlebotomine sand flies. According to the World Health Organization (WHO), it is classified as a neglected tropical disease [1].

The disease accounts for approximately 1.3 million cases and up to 30,000 deaths annually, and it is mainly spread in the tropical and subtropical regions [2]. Depending on the infecting *Leishmania* species and host immune factors, the disease is divided into three clinical forms: cutaneous, mucocutaneous, and visceral leishmaniasis (VL) (also known as kala-azar) [1]. The disease exhibits a broad clinical spectrum, extending from self-limiting cutaneous ulcers to mucocutaneous clinical form with cartilage involvement and destruction, to the life-threatening visceral form [3].

VL is mostly caused by *Leishmania donovani* (*L. donovani*) and *Leishmania infantum* (*L. infantum*) [4]. *L. infantum* is the most common autochthonous species that causes visceral leishmaniasis in Europe. Only rare cases of *L. donovani sensu stricto* transmission were reported in Europe, in Cyprus [5]. The primary domestic infection reservoir for *L. infantum* is dogs, whereas humans are considered an incidental host [5].

In humans, VL typically presents with fever, weight loss, pallor, pancytopenia, and hepatosplenomegaly, carrying a mortality rate of up to 10%, particularly in immunocompromised patients. While most infections remain asymptomatic and result in long-term immunity, active disease develops when the parasite evades host defenses to proliferate within the reticuloendothelial system [6,7]. Parasite survival within the host is facilitated by mechanisms that interfere with inactivation of complement pathways, impairment of macrophage-derived reactive oxygen and nitrogen species, and downregulation of antigen-specific CD4+ T helper cell responses [4]. This replication induces organ hyperplasia and a severe cytokine-mediated systemic inflammatory response [6]. Advanced VL is characterized by severe cachexia, hypoalbuminemia, and edema. Ascites, jaundice, and hepatic dysfunction can develop later in the course of the disease. Hepatic dysfunction and thrombocytopenia are two factors that cause hemorrhagic complications. Individuals may have spontaneous bleeding from the nasal mucosa, gingiva, or various other places. In rare cases, intestinal parasite invasion might result in persistent diarrhea and malabsorption [4].

The diagnosis of VL is complicated by its non-specific clinical presentation, which mimics diseases such as malaria and tuberculosis, and by the sequestration of parasites within the reticuloendothelial system. The gold standard remains direct observation of the parasite by microscopy (Giemsa staining) or culture from invasive samples, most commonly bone marrow aspirates in Europe, Brazil, and the United States and splenic aspirates in East Africa and the Indian subcontinent. Correct identification requires qualified personnel, which can be a limiting factor in countries where VL is rarely documented [8].

Although splenic aspiration offers higher sensitivity, it carries a substantial risk of severe hemorrhage, particularly in patients with advanced disease or coagulation disorders, making bone marrow aspiration the safer and more practical alternative despite its lower sensitivity and greater patient discomfort [9,10].

Serological tests are a cost-effective option in immunocompetent patients, although their accuracy varies with endemic context, antigen, and the patient’s age and immune status [11].

Among molecular methods, PCR assays amplifying parasite DNA provide high sensitivity and specificity, with performance depending on the biological sample and primers used [12].

The epidemiology and ecology of VL are shaped by parasite species, sand fly vectors, and mammalian reservoir hosts [4]. In Europe, VL is largely indigenous, with approximately 88% of infections acquired within European borders [13]. Between 2005 and 2008, the WHO-GHDR reported median annual VL incidences per 100,000 population ranging from 3.8 in Albania to 0.12–0.24 in Italy, Portugal, and Croatia, with Spain at 0.51 and intermediate values in Montenegro, Greece, and North Macedonia (0.33–0.41) [14,15]. More recently, Maia et al. analyzed WHO-GHO data spanning 2005–2020 and reported the highest cumulative autochthonous VL incidence in Albania (2.15), followed by Montenegro, Malta, Greece, Spain, and North Macedonia (0.53–0.42), Italy (0.16), and Portugal (0.09). Hospital discharge data suggested higher actual incidences than those reported to the WHO (0.70 in Italy, 0.67 in Spain, 0.41 in Portugal), and underreporting remains substantial. No widespread increase in autochthonous VL incidence was identified, although declining trends were noted in Albania, Italy, and Portugal, with peaks in Greece (2013, 2014, 2017) and Spain (2006–2007, 2011–2013) [5]. According to the most recent WHO surveillance update including data up to 2023, 53 of the VL-endemic countries (66%) reported to the WHO Global Leishmaniasis program, with the global burden concentrated in seven countries: Brazil, Ethiopia, India, Kenya, Somalia, South Sudan, and Sudan [16].

While *Leishmania* spp. circulation is well-documented in Southern and Eastern Romania, its history in Western Romania is predominantly characterized by imported pathology rather than local transmission [17,18,19,20]. Retrospective analyses have previously identified sporadic imported cases in Timișoara, specifically among migrant workers returning from endemic Mediterranean regions such as Spain in the mid-2000s [21]. However, the medical literature currently lacks data on imported VL in this specific region. Here, we report two cases of VL diagnosed in Timiș County, both imported from endemic areas of eastern Spain: one with a classic clinical presentation and one with an atypical, oligosymptomatic presentation.

## 2. Case Presentation

### 2.1. Case 1

A 57-year-old male patient from Timiș County, Western Romania, presented to the Emergency Department of the Timișoara Municipal Emergency Clinical Hospital in June 2020 with a three-month history of progressive asthenia, fatigue, and profuse nocturnal sweating. He reported unintentional weight loss of approximately 8 kg over the preceding month and a recent episode of rectorrhagia. His past medical history included ischemic heart disease, Type 2 Diabetes Mellitus under oral antidiabetic treatment and past Hepatitis B infection. Notably, the patient disclosed a travel history to Spain (Castellón region) 9 and 24 months prior to presentation.

Physical examination and initial imaging (computed tomography) revealed significant splenomegaly (spleen dimensions 14.8/8.2 cm) with homogeneous structure, hepatomegaly with steatosis, and mediastinal lymphadenopathy. Laboratory investigations at admission demonstrated severe pancytopenia (leukocytes 1.5 × 10^3^/µL, hemoglobin 8.7 g/dL, platelets 84 × 10^3^/µL) and a marked inflammatory syndrome (erythrocyte sedimentation rate (ESR) 140 mm/h, C-reactive protein (CRP) 48 mg/L, ferritin 890 ng/mL). Immunoelectrophoresis indicated polyclonal hypergammaglobulinemia (IgG 39.4 g/L). Testing for Severe Acute Respiratory Syndrome Coronavirus 2 (SARS-CoV-2) by Reverse Transcription Polymerase Chain Reaction (RT-PCR) was negative.

**Figure 1:**
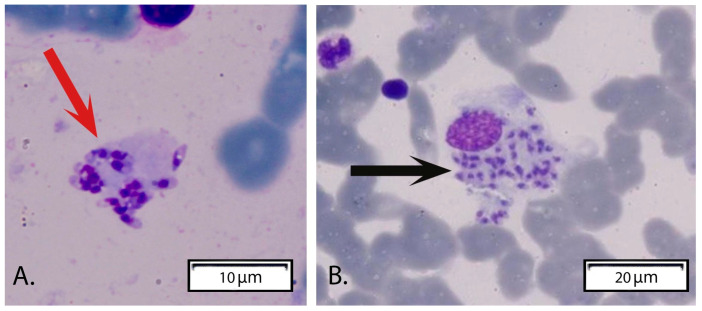
A detailed view of extracellular *Leishmania* amastigotes (**red arrow**) (**A**) and within medullar macrophages (**black arrow**) (**B**) found in the May–Grunwald–Giemsa-stained bone marrow aspirate of case 1.

Due to the persistence of pancytopenia and fever of unknown origin, a bone marrow aspiration was performed at the Hematology Department of the Timișoara Municipal Emergency Clinical Hospital. Cytological examination of the smear revealed massive infiltration (grade 6+) with intracellular and extracellular *Leishmania* amastigotes (Leishman-Donovan bodies), primarily within macrophages, as well as free-flowing organisms (Figure 1).

**Figure 2:**
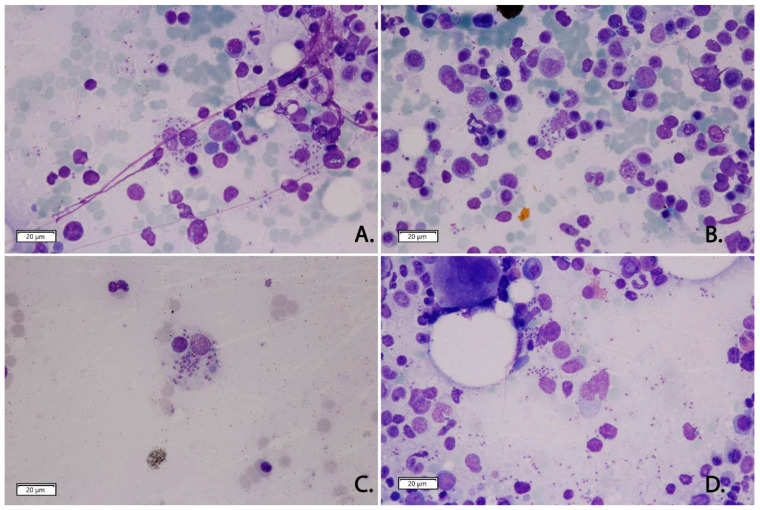
An overview of the May–Grünwald–Giemsa-stained bone marrow aspirate smears demonstrating hypercellular marrow (**A**,**B**,**D**) with abundant *Leishmania* amastigotes identified both intracellularly within macrophages (**A**–**C**) and extracellularly (**D**) in case 1.

A general overview of the bone marrow infiltration is presented in Figure 2. The smear was initially analyzed in the laboratory of Timișoara Municipal Emergency Clinical Hospital, with the findings further confirmed at the Center for Diagnosis and Study of Parasitic Diseases, Department of Parasitology, Victor Babes University of Medicine and Pharmacy. The formol-gel test was positive within 5 min. Subsequent serological testing further supported the diagnosis, returning positive for anti-*Leishmania* IgG antibodies.

The case was classified as VL, likely imported given the travel history to an endemic region in Spain, although the latency period suggests a chronic or reactivated course. Due to the unavailability of specific anti-leishmanial medication (e.g., liposomal amphotericin B) at the admitting hematology unit, the patient received supportive care, including broad-spectrum antibiotics (ceftazidime) and anticoagulation, and was subsequently referred to a specialized Infectious Diseases clinic for definitive therapy.

### 2.2. Case 2

The second case consisted of a 51-year-old male patient from a rural area in Timiș County (Cărpiniș), Western Romania, who presented to the hematology outpatient clinic of the Timișoara Municipal Emergency Clinical Hospital in July 2020 complaining of marked physical asthenia and fatigue. His past medical history was significant for Psoriatic Arthritis, Essential Hypertension (Grade II), and chronic venous insufficiency with post-thrombotic syndrome of the left lower limb. The patient reported a recent travel history to the València region of Spain, a known endemic area for VL.

Upon admission to the Hematology clinic, physical examination revealed pale skin and mucous membranes and multiple cutaneous lesions consistent with psoriasis. Notably, unlike the classical presentation of VL, the patient did not present with palpable superficial lymphadenopathy or organomegaly (hepatosplenomegaly) at the time of clinical examination; the abdomen was described as distended but without palpable masses. The patient was afebrile.

Biochemical and hematological investigations conducted at the Clinical Laboratory of Timișoara Municipal Emergency Clinical Hospital revealed bicytopenia characterized by moderate hypochromic microcytic anemia (hemoglobin 9.6 g/dL, mean corpuscular volume (MCV) 76.5 fL) and leukopenia (white blood cell count (WBC) 2.43 × 10^3^/µL) with moderate neutropenia (neutrophils 1.00 × 10^3^/µL). Platelet counts were within normal limits (188 × 10^3^/µL). An intense inflammatory syndrome was observed, with an extremely elevated ESR of 160 mm/h, CRP of 59.4 mg/L, and ferritin levels of 624.2 ng/mL. Hepatic and renal function tests were largely unremarkable (alanine aminotransferase (ALT) 13 U/L, aspartate aminotransferase (AST) 26 U/L, creatinine 1.09 mg/dL). Screening for human immunodeficiency virus (HIV), Hepatitis B, Hepatitis C, and SARS-CoV-2 returned with negative test results.

**Figure 3:**
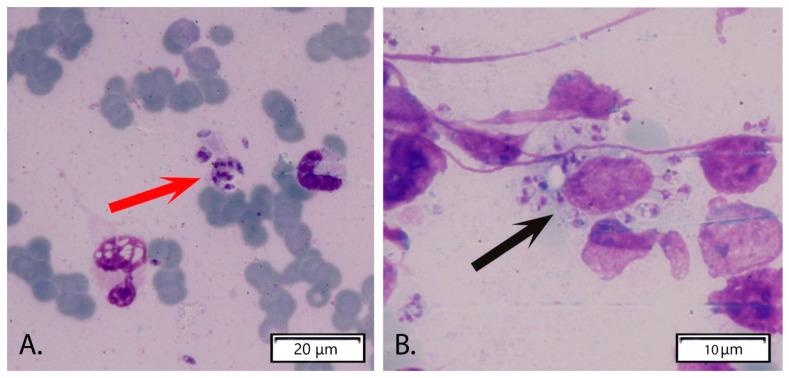
A detailed view of extracellular *Leishmania* amastigotes (**red arrow**) (**A**) and within medullar macrophages (**black arrow**) (**B**) found in the May–Grunwald–Giemsa-stained bone marrow aspirate of case 2.

Given the unexplained bicytopenia and inflammatory syndrome, a sternal bone marrow aspiration was performed one day after initial presentation in the Hematology Department of the Timișoara Municipal Emergency Clinical Hospital. The sample was analyzed at the Clinical laboratory of the same hospital. The bone marrow aspirate examination revealed a hypercellular marrow (“rich cellularity”) with massive infiltration of parasitic formations. Microscopic analysis identified numerous intracellular (predominantly within macrophages) and extracellular amastigote forms, morphologically consistent with *Leishmania* spp. (Figure 3).

**Figure 4:**
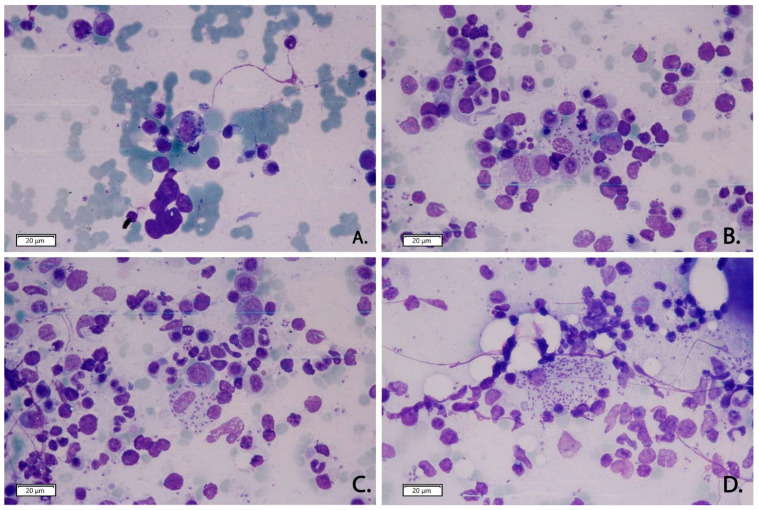
An overview of the May–Grünwald–Giemsa-stained bone marrow aspirate smears demonstrating hypercellular marrow (**B**–**D**) with abundant *Leishmania* amastigotes identified both intracellularly within macrophages (**A**–**C**) and extracellularly (**D**) in case 2.

Figure 4 presents an extended overview of the examined bone marrow aspirate. The findings were also confirmed at the Center for Diagnosis and Study of Parasitic Diseases, Department of Parasitology, Victor Babes University of Medicine and Pharmacy.

The patient was diagnosed with VL with bone marrow invasion. Following the confirmation of the parasitic etiology, the patient was discharged with a recommendation for immediate transfer to a specialized Infectious Diseases and Tropical Medicine clinic for specific serological confirmation (Anti-*Leishmania* IgG) and initiation of targeted therapy.

A comparison of the two cases presented in this report can be found in Table 1.

## 3. Discussion

In Europe, leishmaniasis is a serious public health issue since it is prevalent in many places and has lately been discovered in regions where it was never reported before [22].

Ready (2010) highlighted that the main threat of leishmaniasis emergence in Europe comes from the northward spread of *L. infantum* from the Mediterranean region, driven by climate change, vector expansion, and increasing dog travel, rather than from the introduction of exotic *Leishmania* species [23].

In a multi-center retrospective analysis of 1142 leishmaniasis cases across 15 European centers, Van der Auwera et al. (2022), found that VL was predominantly acquired within Europe (88%), reinforcing the need for integrated cross-border surveillance to monitor changing epidemiology in both endemic and non-endemic countries [13]. In neighboring Bulgaria, Vutova et al. (2024) reported clinical data from 58 VL patients treated over a 45-year period, highlighting that the disease is primarily endemic in the southern regions and that diagnostic delays of up to 28 months remain a significant challenge [24].

Mihalca et al. (2019) reviewed the epidemiological situation of leishmaniasis across Eastern Europe, noting that while most countries in the region are considered non-endemic, new imported or autochthonous cases in both humans and dogs are being reported with increasing frequency, highlighting the need for sustained surveillance [25]. In Romania, the first documented case of human leishmaniasis was reported by Manicatide (1919-1920) [26]. In the following years, additional isolated cases and outbreaks were reported. However, all of them were documented in the southern part of the country [25].

In 2009, Neghina et al. reported three cases of imported VL in Romanian workers who returned from Spain to Timișoara in 2005. Researchers highlighted the importance of correlating the clinical syndrome with travel history and occupational exposure in endemic Mediterranean regions [21].

In 2010, Găman et al. (2010) described a case of VL in Dolj county, in a patient who returned from a fourteen-month stay in Greece [27]. In 2013, Gogoașe et al. (2013) reported two cases of VL caused by *L. donovani complex* diagnosed at Fundeni Clinical Institute, in which autochthonous transmission in southwestern Romania could not be excluded [28]. One year later, Alexa et al. (2014) reported a case of VL in a 44-year-old female from Iasi county with a history of working in Italy [19]. In 2025, Mihu et al. reported a 25-year-old female kidney transplant recipient with VL in Cluj County [17]. In the present report, both cases originated from Timiș County, Western Romania, and both patients reported recent travel to endemic regions of eastern Spain (Castellón and València) prior to hospital presentation.

The first patient presented with fever, weight loss and rectorrhagia while the second case reported fatigue and asthenia. Usually, VL requires a period of two to eight months to manifest. The main symptoms reported in VL include fever, night sweats, weight loss, weakness, and loss of appetite [2]. Rectal bleeding is not a common presentation in VL. However gastrointestinal bleeding was documented in several cases [29,30].

Clinical examination revealed marked splenomegaly and hepatomegaly in the first case, while the second patient did not present any organ enlargement. Usually, clinical examination of patients with VL may reveal pallor, enlargement of the liver and spleen and enlarged lymph nodes [2]. Hepatosplenomegaly is one of the most significant diagnostic indicators for VL. Histopathological examinations have indicated that the spleen and liver are loaded with parasites, indicating lymphoid hyperplasia. There is no evidence that passive hypertensive congestion causes splenomegaly. The increase in spleen volume is due to an accumulation of parasite-containing macrophages and plasma cell hyperplasia, despite a noticeable reduction in the splenic white pulp linked to necrosis and fibrosis of thymus-dependent regions. Proinflammatory and regulatory cytokine production in infected people and dogs is linked to the quantity of parasites in the spleen and the disturbance of its architecture [6].

In a landmark review of 47 VL cases among patients with HIV, Peters et al. found that splenomegaly, nearly universal in immunocompetent hosts, was absent in eight (17%) [31]. Pintado et al. confirmed this in a larger comparative series, reporting splenomegaly in only 80.8% of HIV-positive patients versus 97.4% of HIV-negative individuals (*p* = 0.02) [32]. Atypical presentations are not limited to HIV coinfection; Kritikos et al. described VL without either hepatomegaly or splenomegaly in a 77-year-old patient on anti-TNF-α therapy (infliximab) for rheumatoid arthritis [33]. Even among immunocompetent patients, VL may lack its hallmark features: Mohan et al. (2007) reported an 84-year-old man presenting solely with severe anemia and heavy parasitemia, in the absence of both fever and splenomegaly [34], while Dehghani et al. (2019) described an 18-year-old immunocompetent patient with VL presenting exclusively with pulmonary consolidation and lymphadenopathy, with an entirely normal abdomen and no organomegaly, diagnosed only by fine needle aspiration of pulmonary nodules revealing Leishman bodies [35].

Regarding the paraclinical examination results, first patient presented severe pancytopenia while the second had bicytopenia. Both cases had high values of the inflammatory markers. Hematological profile dysregulation has been associated with VL patients, and this might be an important contributor to mortality and morbidity [36]. In a study from Ethiopia, the prevalence of anemia, leukopenia, and thrombocytopenia in patients with VL was 85.5%, 83.4%, and 75.8% [36]. VL has been associated with pancytopenia and bicytopenia [17].

Pancytopenia means a reduction in all three major blood cell lineages, while bicytopenia is defined as a decrease in any two of the three major blood cell lineages: leukocytes, erythrocytes, or platelets. Because of systemic and splenic inflammation, which promotes the mechanical destruction of blood cells and platelets, cytopenias are usually more severe in VL, even though they also occur in noninflammatory hypersplenism [6,17,37,38,39]. The important role of inflammation is supported by the rapid and complete resolution of leukopenia and thrombocytopenia following splenectomy in relapsing VL, compared to only partial recovery in noninflammatory hypersplenism. Anemia in VL is also caused by inflammation, with hepcidin playing a major role in the acute-phase response, resulting in functional iron restriction. Findings from bone marrow reveal erythroid hyperplasia and dysplasia instead of hypoplasia, suggesting that splenic sequestration and destruction of red blood cells, exacerbated by inflammation-associated iron limitation, rather than compromised marrow production, are the primary causes of anemia [6,40].

In both of the cases, inflammatory markers were above the normal limit. Proteins whose concentration in peripheral blood increase by more than 25% during inflammation are referred to be acute phase proteins. Both infectious and non-infectious inflammatory processes, as well as malignant conditions, raise their serum levels [41,42]. A study in Sudan analyzed the association between CRP and the development of post kala-azar dermal leishmaniasis. Patients with moderately elevated CRP (up to 30 mg/L) had a low risk of developing Post-kala-azar dermal leishmaniasis (PKDL) after treatment, whereas patients with high CRP levels (over 40 mg/L) had a very high risk of PKDL [42].

Diagnosis in both patients was based on serology and confirmed through identification of amastigote forms in bone marrow aspirates. Several immunodiagnostic techniques that are both sensitive and specific have been developed. They are valuable for identifying individual cases and can be used to monitor communities. In an attempt to combat VL, the human body produces perhaps one of the greatest quantities of antibodies identified in response to any illness, but to no effect. Immunoglobulin G (IgG) and IgM levels (in serum) against a variety of nonspecific proteins and haptens are markedly elevated as a result of polyclonal activation of the B cells. The constant presence of high levels of antibodies against parasite antigens helps facilitate the diagnosis of VL [43].

Given recent data and documented cases in this region of Europe (Maia et al., 2023 [5]; Ready, 2010 [23]), the emergence of autochthonous leishmaniasis should be considered a realistic possibility in the near future [44,45,46]. The potential for autochthonous VL transmission in Romania is increasingly supported by data from entomological, climatic, and epidemiological research [5,45]. Of the eight phlebotomine sand fly species historically recorded in the country, three, *Phlebotomus* (*Ph.*) *perfiliewi*, *Ph. neglectus*, and *Ph. balcanicus*, are known vectors of *L. infantum* [47].

A nationwide survey (2013–2018) confirmed five species across southwestern, eastern, and southern Romania, with Ph. neglectus predominating; notably, their current distribution differs from historical records and overlaps with areas of Mediterranean climatic influence [47]. Entomological investigations have also found sand fly populations in Mehedinți County in southwestern Romania, which is geographically near to Timiș County, adding credibility to local transmission’s ecological possibility. Of relevance, *Ph. perfiliewi* was documented in northeastern Romania with defined seasonal dynamics, demonstrating that competent vectors are established well beyond the traditional southern foci [48]. Climate modeling studies project a northward expansion of suitable habitats for several *Phlebotomus* species, with Romania and the Carpathian Basin becoming increasingly favorable [49,50,51]. This trend was recently confirmed by a European-wide analysis showing increased climatic suitability for leishmaniasis in southern and eastern countries, including Romania [52]. Similar concerns have been raised across the Mediterranean basin, where environmental and climatic transformations are shifting sand fly distributions northward into previously non-endemic areas [53].

The zoonotic nature of *L. infantum* is also an important factor, with domestic dogs serving as the primary reservoir of infection. The first clinical case of autochthonous canine leishmaniasis in Romania in over 80 years was reported in 2014 in a dog from Vâlcea County with no travel history [44]. A follow-up study in the same area detected *L. infantum* DNA in 8.7% of the surveyed dogs, all sub clinically infected, confirming a local transmission focus [45]. These findings are consistent with a broader historical review suggesting low-level enzootic transmission that has long been neglected or underdiagnosed in Romania [25]. Although both cases reported here were imported from Spain, the presence of competent vectors, documented canine infections, and favorable climatic trends suggest that Western Romania may become increasingly susceptible to autochthonous transmission in the future, underscoring the need for integrated One Health surveillance.

This report has limitations. First, it is based on only two cases, which precludes any generalizable conclusions regarding the epidemiology, clinical spectrum, or treatment outcomes of VL in Romania or in Europe more broadly. Second, species-level identification of the parasite by molecular methods (e.g., PCR-based typing) was not performed, and the etiological assignment to *L. infantum* is therefore inferred from the geographic origin of exposure rather than confirmed at the molecular level. Nevertheless, this inference is supported by the fact that *L. infantum* is the sole autochthonous *Leishmania* species described in Spain, where it is responsible for both visceral and cutaneous forms of the disease in humans [54,55]. Third, follow-up data after referral to specialized infectious disease units were not available, limiting our ability to comment on long-term outcomes. Despite these limitations, we believe the report retains its value as an awareness-oriented contribution, intended to support timely recognition of imported visceral leishmaniasis in non-endemic regions.

## 4. Conclusions

This report describes two imported cases of VL in Timiș County, Western Romania, both linked to endemic areas of eastern Spain (Castellón and València), highlighting the diagnostic challenge posed by atypical and oligosymptomatic presentations. These cases illustrate how cross-border travel to endemic Mediterranean regions can introduce VL into traditionally non-endemic areas of Europe, making a thorough travel history essential in the diagnostic workup. The findings underscore the need for heightened clinical suspicion for VL in patients with unexplained cytopenias and inflammatory syndrome in non-endemic European areas, and for clinician awareness of imported leishmaniasis in individuals returning from endemic regions of southern Europe.

## Figures and Tables

**Table 1:** Characteristics of the Visceral Leishmaniasis Cases Diagnosed in Timiș County, Western Romania.

Characteristics	Case 1	Case 2
Demographics	Male, 57 years old	Male, 51 years old
Origin	Western Romania (Timiș County)	Western Romania (Timiș County)
Travel History	Yes (Spain: Castellón region)	Yes (Spain: València region)
Comorbidities	Type 2 Diabetes, Ischemic Heart Disease, Hepatitis B	Psoriatic Arthritis, Hypertension
Primary Symptoms	Fever, weight loss, rectorrhagia	Fatigue, asthenia (pauci-symptomatic)
Organomegaly	Massive Splenomegaly (14.8 cm), Hepatomegaly	Absent (No palpable organomegaly)
Hematology	Severe Pancytopenia	Bicytopenia
	(WBC 1.5 × 10^3^/µL, Plt 84 × 10^3^/µL)	(WBC 2.43 × 10^3^/µL, Plt normal)
Inflammatory Markers	High (Ferritin 890 ng/mL)	High (Ferritin 624 ng/mL, ESR 160 mm/h)
Diagnosis Method	Bone Marrow Aspirate (Amastigotes present)	Bone Marrow Aspirate (Amastigotes present)
Clinical Classification	Classic Visceral Leishmaniasis	Atypical/Oligosymptomatic Visceral Leishmaniasis

## Data Availability

The original contributions presented in this study are included in the article. Further inquiries can be directed to the corresponding authors.

## Abbreviations

The following abbreviations are used in this manuscript:

ALT	Alanine aminotransferase
AST	Aspartate aminotransferase
CRP	C-reactive protein
ESR	Erythrocyte sedimentation rate
HIV	Human immunodeficiency virus
IgG	Immunoglobulin G
IgM	Immunoglobulin M
*L. donovani*	*Leishmania donovani*
*L. infantum*	*Leishmania infantum*
MCV	Mean corpuscular volume
PCR	Polymerase chain reaction
Spp.	Species
PKDL	Post-kala-azar dermal leishmaniasis
RT-PCR	Reverse Transcription Polymerase Chain Reaction
SARS-CoV-2	Severe Acute Respiratory Syndrome Coronavirus 2
VL	Visceral Leishmaniasis
WBC	White blood cell count
WHO-GHDR	World Health Organization–Global Health Data Repository
